# Supplementary material for: Genome-Wide Identification of the Ginkgo (Ginkgo biloba L.) LBD Transcription Factor Gene and Characterization of Its Expression
Source: Int J Mol Sci. 2022 May 13;23(10):5474. doi: 10.3390/ijms23105474 (PMC9141976; doi:10.3390/ijms23105474)
Supplement: Supplementary file 1 [file ijms-23-05474-s001.zip › ijms-1684834-supplementary.pdf]

International Journal of *Molecular Sciences*

MDPI

Table S1. Ginkgo *LBD* gene characteristics.

Gene Name	Gene ID	Type	Ortholog				Size(aa)	MW(Da)	pI	StabIlIty	Predicted Location
			AtLBD	Identify(%)	PtLBD	Identify(%)					
GbLBD01	**Gb_20398**	**Ia**	***AtLBD15***	**77.50**	***Potri.013G081200***	**73.28**	179	19654.27	8.39	U	Nucleus
GbLBD02	Gb_23794	Ic	-	-	*Potri.001G281600*	66.67	311	34327.78	8.51	U	Nucleus
GbLBD03	Gb_21467	If	-	-	-	-	251	28300.81	7.58	U	Nucleus
GbLBD04	Gb_13424	Ia	-	-	-	-	238	24776.23	8.63	U	Nucleus
GbLBD05	Gb_07877	If	-	-	-	-	362	40996.89	7.24	U	Nucleus
GbLBD06	Gb_19567	Id	-	-	-	-	115	13049.83	8.93	U	Nucleus
GbLBD07	Gb_38978	Ia	*AtLBD15*	74.83	*Potri.013G081200*	84.80	178	19418.17	8.97	U	Nucleus
GbLBD08	Gb_29853	Ic	*AtLBD12*	73.68	*Potri.007G066700*	68.42	176	19171.03	8.18	U	Nucleus
GbLBD09	Gb_14010	Ib	-	-	-	-	288	31232.51	7.17	U	Nucleus
GbLBD10	Gb_22178	Id	-	-	-	-	257	29360.18	6.54	S	Nucleus
GbLBD11	Gb_19384	Id	-	-	-	-	174	19941.43	5.92	U	Nucleus
GbLBD12	Gb_19383	Id	-	-	-	-	179	20286.76	4.99	U	Nucleus
GbLBD13	Gb_19390	Id	-	-	-	-	172	19238.63	5.83	U	Nucleus
GbLBD14	Gb_19389	Id	-	-	-	-	175	19970.24	5.30	U	Nucleus
GbLBD15	Gb_10524	Ia	*AtLBD15*	69.51	*Potri.013G081200*	68.12	197	21639.58	8.94	U	Nucleus
GbLBD16	Gb_10825	IIa	-	-	-	-	324	36227.24	7.80	U	Nucleus
GbLBD17	Gb_40767	Ia	-	-	-	-	189	20743.55	9.05	U	Nucleus
GbLBD18	Gb_19720	If	-	-	-	-	306	34932.94	8.78	U	Nucleus
GbLBD19	Gb_39343	Ib	-	-	*Potri.002G041200*	68.12	223	24724.12	6.14	U	Nucleus
GbLBD20	Gb_16914	Id	-	-	-	-	174	19234.57	4.92	U	Nucleus
GbLBD21	Gb_16918	Id	*AtLBD12*	67.89	-	-	175	19181.45	5.11	U	Nucleus
GbLBD22	Gb_20676	Id	-	-	-	-	186	20709.77	7.65	U	Nucleus
GbLBD23	Gb_36943	IIa	-	-	-	-	515	57684.72	8.91	U	Nucleus
GbLBD24	Gb_36942	IIa	-	-	-	-	420	46037.00	8.88	U	Nucleus
GbLBD25	Gb_21109	Ic	*AtLBD4*	68.02	*Potri.001G08140*	71.76	273	30263.96	9.27	U	Nucleus
GbLBD26	Gb_09463	Id	-	-	-	-	174	19156.48	4.41	U	Nucleus
GbLBD27	Gb_40684	Id	-	-	-		150	17013.35	4.99	U	Nucleus
GbLBD28	Gb_22437	Id	-	-	-	-	197	22136.24	6.09	S	Nucleus
GbLBD29	Gb_36035	Id	-	-	-	-	197	22120.27	5.81	U	Nucleus
GbLBD30	Gb_02917	Id	-	-	-	-	119	13728.72	8.73	U	Nucleus
GbLBD31	Gb_38119	Ic	*AtLBD4*	74.19	*Potri.005G097800*	75.20	136	14800.99	9.08	U	Nucleus
GbLBD32	Gb_03653	Ia	*AtLOB*	68.42	*Potri.012G083500*	73.85	231	25331.83	8.26	U	Nucleus
GbLBD33	Gb_02663	Ib	-	-	-	-	239	25923.15	6.15	U	Nucleus
GbLBD34	Gb_00144	Id	*AtLBD1*	72.32	*Potri.001G281600*	69.72	146	16593.95	6.40	U	Nucleus
GbLBD35	Gb_09641	IIb	-	-	-	-	633	70905.07	9.68	U	Nucleus
GbLBD36	Gb_23688	Ia	*AtLBD15*	70.32	-	-	203	22114.06	8.97	U	Nucleus
GbLBD37	Gb_08465	Id	-	-	*Potri.010G184400*	65.05	178	20162.60	5.09	U	Nucleus

Table S2. Primer sequences.

Primer ID	Forward PCR primer (5′ -3′)	Reverse PCR primer (5′ -3′)
Gb_GAPDH	GGTGCCAAAAAGGTGGTCAT	CAACAACGAACATGGGAGCAT
GbLBD01	TGTGCCCAGGAGTGTCCGTT	CGCCCTCTGGCTCTCTGGTA
GbLBD04	CCCGTCTATGGCTGCGTTGG	GGGCTTGCCTGCTGTGTCAA
GbLBD05	GCTTGGAAGCTTTCACCGGACT	GGCTTGTGTTGAGCCGCAGT
GbLBD07	GCACGTCTGCAAGACCCTGT	GCATTGGAGTTGGCGGGACT
GbLBD16	GGGATGTCCTGTACGACCGCTA	CCCGCTTCCGGCAGATGATG
GbLBD18	CATCTACGCCGCCGTCTTCC	TGCAGCACAAGCCTGACTCG
GbLBD20	CTACGGTTGTGCAGGCGTCA	TCGGAACGCAGGTTGAGAAGC
GbLBD21	TCGCAACCGCACATCGAGTT	GAGCCTTGCAGTGGCTTCGT
GbLBD23	GGCTGTAGCGACTCCTGCAC	TCAATCCAGCGCGCCCATAG
GbLBD24	GCTGCCGAGTCCTCCGAAAG	GCCCTGCTCGCCCATAGAAC
GbLBD25	GTTGGCCGTTAGCCGGAGTG	ATGCACCGGCCATGAACCAA
GbLBD31	GCCAAGGACTGCGTGTTTGC	CCCTCTCTGCTCCACCGGAA
GbLBD32	TCTGCAGCACCAGGTCAAGC	CGCGAGGTTCCTGCCAATCC
GbLBD33	GCTCTCCAACAACAGGTCGCA	GCAGCAGCAGCAGACGAAGA
GbLBD35	CGCAAGGTCACGCCACTGTA	CCCGCATGCCTCGTACAACA

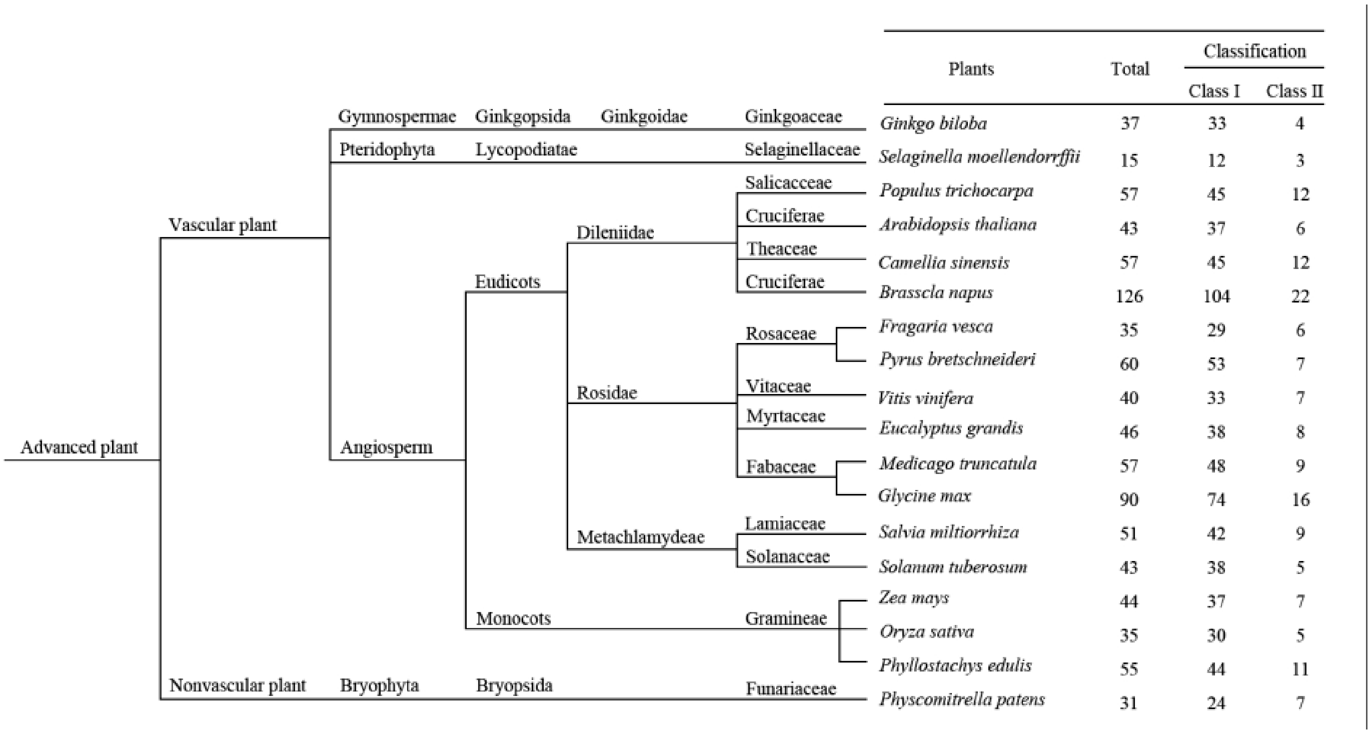

Figure S1. Number of LBD genes in different species.

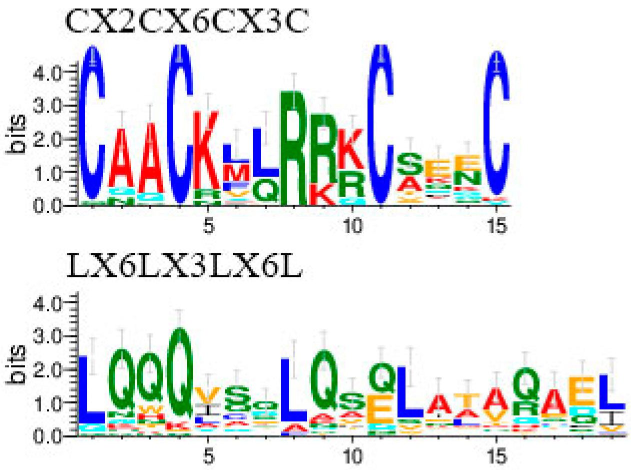

Figure S2. Conserved motif icon of GbLBD protein.
